# The relation between sarcomere energetics and the rate of isometric tension relaxation in healthy and diseased cardiac muscle

**DOI:** 10.1007/s10974-019-09566-2

**Published:** 2019-11-19

**Authors:** Giulia Vitale, Cecilia Ferrantini, Nicoletta Piroddi, Beatrice Scellini, Josè Manuel Pioner, Barbara Colombini, Chiara Tesi, Corrado Poggesi

**Affiliations:** Department of Experimental and Clinical Medicine, University of Florence, Viale Morgagni 63, 50134 Florence, Italy

**Keywords:** Muscle mechanics, Muscle energetics, Cardiac muscle, Myofilaments, Myosin, Troponin, Hypertrophic cardiomyopathy

## Abstract

Full muscle relaxation happens when [Ca^2+^] falls below the threshold for force activation. Several experimental models, from whole muscle organs and intact muscle down to skinned fibers, have been used to explore the cascade of kinetic events leading to mechanical relaxation. The use of single myofibrils together with fast solution switching techniques, has provided new information about the role of cross-bridge (CB) dissociation in the time course of isometric force decay. Myofibril’s relaxation is biphasic starting with a slow seemingly linear phase, with a rate constant, slow *k*_REL_, followed by a fast mono-exponential phase. Sarcomeres remain isometric during the slow force decay that reflects CB detachment under isometric conditions while the final fast relaxation phase begins with a sudden give of few sarcomeres and is then dominated by intersarcomere dynamics. Based on a simple two-state model of the CB cycle, myofibril slow *k*_REL_ represents the apparent forward rate with which CBs leave force generating states (*g*_app_) under isometric conditions and correlates with the energy cost of tension generation (ATPase/tension ratio); in short slow *k*_REL_ ~ *g*_app_ ~ tension cost. The validation of this relationship is obtained by simultaneously measuring maximal isometric force and ATP consumption in skinned myocardial strips that provide an unambiguous determination of the relation between contractile and energetic properties of the sarcomere. Thus, combining kinetic experiments in isolated myofibrils and mechanical and energetic measurements in multicellular cardiac strips, we are able to provide direct evidence for a positive linear correlation between myofibril isometric relaxation kinetics (slow *k*_REL_) and the energy cost of force production both measured in preparations from the same cardiac sample. This correlation remains true among different types of muscles with different ATPase activities and also when CB kinetics are altered by cardiomyopathy-related mutations. Sarcomeric mutations associated to hypertrophic cardiomyopathy (HCM), a primary cardiac disorder caused by mutations in genes encoding sarcomeric proteins, have been often found to accelerate CB turnover rate and increase the energy cost of myocardial contraction. Here we review data showing that faster CB detachment results in a proportional increase in the energetic cost of tension generation in heart samples from both HCM patients and mouse models of the disease.

## Introduction

The mechanical performance of cardiac muscle results from the interplay between two macromolecular systems: (1) membrane bound-calcium handling proteins, responsible for the Ca^2+^ signal that starts and stops contraction; (2) sarcomeric proteins, responsible for active and passive force generation and for contraction regulation by Ca^2+^. To dissect the relative roles of these two systems in the extent and kinetics of striated muscle contraction and relaxation, disparate preparations at all levels of the structural hierarchy have been used.

Though most models about the biochemical steps of crossbridge (CB) cycle and their regulation are based on studies of proteins in solution, isolated proteins lack the structural complexity and mechanical constraints of muscle fibers. Myofibrils, formed by serially arranged sarcomeres, maintain a complete structured ensemble of contractile and Ca^2+^ regulatory proteins. Thanks to the rapid equilibration with the medium, myofibrils allow the investigation of CB kinetic processes (for reviews see Poggesi et al. 2005; Stehle et al. 2009; Stehle and Tesi 2017). The possibility to easily obtain myofibrils from small human cardiac samples also provide a useful model to study disease-related mechanical and kinetic dysfunction in human cardiac sarcomeres (Neagoe et al. 2002; Piroddi et al. 2007, 2019; Belus et al. 2008, 2010; Walker et al. 2011; Witjas-Paalberends et al. 2014a; Vikhorev et al. 2017; Jeong et al. 2018).

Familial hypertrophic cardiomyopathy (HCM) is the most common inherited heart disease with a prevalence of 1:500 (Maron et al. 2014). It is considered a disease of the sarcomere because several genes encoding cardiac myofilament proteins have been shown to cause the disease (Watkins et al. 2011; Poggesi and Ho 2014). Some HCM-associated sarcomeric mutations primarily result in aberrant/faster CB dynamics and increased ATP usage for the force generation process (Belus et al. 2008; Witjas-Paalberends et al. 2014a; Ferrantini et al. 2017; Piroddi et al. 2019). It has been suggested long ago that sustained energy impairment in cardiomyocytes may be central in HCM disease (Crilley et al. 2003; Javadpour et al. 2003; Ashrafian et al. 2003).

Since the origin of the coupling between mechanic and energetic events during cardiac sarcomere contraction resides in the rates governing CB turn over, myofibril mechanic experiments associated with mechanic and energetic experiments in skinned muscle strips, represent unique tools to dissect the physiology and pathophysiology of sarcomere dynamics and energetics.

### Myofibrils and fast solution switching: experimental tools to investigate apparent CB kinetics

While the complete cellular physiology of muscle contraction and relaxation can be only studied in intact muscle preparations, dissecting the roles played by CBs and myofilament regulation in the kinetics of the twitch is obscured by the relatively slow rates of myoplasmic Ca^2+^ rise and removal (Caputo et al. 1994; Gao et al. 1998; Bers 2001). To focus on the process of force generation and relaxation and its regulation at the sarcomere level, skinned muscle preparations are to be employed. Indeed, the removal of all plasmatic and Sarcoplasmic Reticulum membranes allows investigators to experimentally control the [Ca^2+^] in the surrounding solution and focus on the process of force generation and relaxation at myofilament level. Caged Ca^2+^-compounds and caged Ca^2+^-chelators can be used in skinned cardiac muscle preparations to abruptly change the [Ca^2+^] by flash photolysis and dissect the myofilament mechanisms of force activation and force relaxation from those related to myoplasmic Ca^2+^ handling (e.g. Palmer and Kentish 1998). The use of caged compounds, however, is not free from problems. Caged compounds have general inherent limitations with respect to the input of large radiant energies and the production of reactive photolytic by‐products as well as uncertainties in the actual concentration of the compounds inside the skinned muscle preparations. Moreover, the kinetics of force activation and relaxation require different caged compounds and must be studied separately (see Palmer and Kentish 1998). If we want to focus on the relaxation process one significant additional limit of caged compounds is that the increased Ca^2+^ buffering capacity following photolysis of caged Ca^2+^-chelators is not enough to induce a complete relaxation from high levels of activation (Wahr et al. 1998; Luo et al. 2002).

Isolated myofibrils, as a model for mechanical experiments, hold some advantages over larger preparations and are ideal to obtain insight about the CB kinetics during an activation-relaxation cycle (e.g. Poggesi et al. 2005). Isolated myofibrils are the smallest subdivision of the contractile apparatus of striated muscle that retain the organized filament lattice and entire ensemble of associated proteins. Because of their small size (the diffusion distances are < 2 µm), rapid (< 1 ms) equilibration is ensured with surrounding solution and thus, the myofilament lattice can be effectively clamped at any desired composition by flowing streams of solution over the preparation. Rapid solution switching methods in myofibrils have given straightforward insights into the kinetics of force activation and relaxation following abrupt increase and decrease of [Ca^2+^] (for a review see Stehle et al. 2009).

**Fig. 1:**
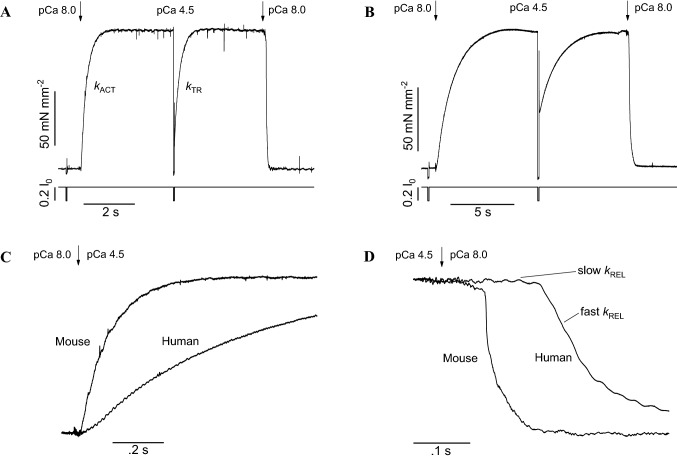
Tension activation and relaxation of mouse and human ventricular myofibrils. **A**, **B** Top traces, representative tension records of mouse (**A**) and human (**B**) ventricular myofibrils maximally Ca^2+^-activated and fully relaxed by fast solution-switching (15 °C). Resting sarcomere length 2.2 μm. The arrows mark the start of the solution changes that modify the myofibril pCa as indicated. Bottom traces are length signals showing the release-restretch protocol applied to the myofibrils under conditions of steady Ca^2+^-activation. *k*_ACT_ is the rate constant of tension development following maximal Ca^2+^ activation; *k*_TR_ is the rate constant of tension redevelopment following release-restreach protocol during steady-state Ca^2+^ activation. **C**, **D** Same traces as in **A**, **B** superimposed on a faster time base after normalization for maximal tension to highlight force activation (**C**) and relaxation (**D**) kinetics. As shown in **D** full tension relaxation from maximal activation is biphasic both in mouse and human myofibrils. The rate constant of the early slow force decline (slow *k*_REL_) is estimated from the slope of the regression line fitted to the force trace normalized to the entire amplitude of the force relaxation transient. The rate constant for the final fast phase of tension decline (fast *k*_REL_) is estimated from mono-exponential fit. Of note, both the kinetics of force activation (**C**) and force relaxation (**D**) are much faster in the mouse compared to the human ventricular myofibril indicating faster cross-bridge turnover and faster detachment rate respectively

The abrupt rise of [Ca^2+^] by fast solution switching in human and mouse ventricular myofibrils at optimum overlap (see Fig. 1A, B) initiates tension development processes that are qualitatively similar but with much different rates. At maximal Ca^2+^ activation, tension rises approximately mono-exponentially, with a rate constant *k*_ACT_ that is one order of magnitude faster in the mouse ventricular myofibril compared to the human preparation (Fig. 1C). Under steady-state conditions of force generation a large mechanical perturbation is applied to the myofibrils aimed at detaching most of the attached force generating CBs (Fig. 1A, B). This mechanical perturbation drops force to zero before starting a force redevelopment process, with rate *k*_TR_, that is thought to reflect the apparent CB turnover rate under conditions of steady-state activation (Brenner 1988). Under our usual experimental conditions, *k*_ACT_ is the same as *k*_TR_, indicating that thin filament Ca^2+^-activation is a rapid process and that both kinetic parameters reflect the apparent rate of CB turn-over.

Full force relaxation of human and mouse ventricular myofibrils, induced by rapid reduction in [Ca^2+^] below contractile threshold, is markedly biphasic (Fig. 1D), like in all myofibril types studied so far. The behaviour parallels that previously described in intact skeletal muscle fibres relaxing from maximum tetanic force (Huxley and Simmons 1970, 1973; Cleworth and Edman 1972; Edman and Flitney 1982). Force relaxation starts with a slow, seemingly linear, phase with a rate constant slow *k*_REL_ that is thought to reflect the apparent rate of CB detachment under isometric conditions also because in myofibril experiments the bulk of the slow relaxation phase occurs after [Ca^2+^] has been effectively reduced below contractile threshold (Poggesi et al. 2005). The slow force decay is followed by a rapid and exponential decay of force, with a rate constant fast *k*_REL_. While myofibril sarcomeres remain isometric during the early slow relaxation phase, the final fast force decay is initiated when a few, mechanically weaker, sarcomeres rapidly lengthen (sarcomere “give”) and is then dominated by inter-sarcomere dynamics (Stehle et al. 2002; Poggesi et al. 2005). Overall force relaxation is much faster in the mouse compared to the human preparation (Fig. 1D).

Previous studies in myofibrils have shown that CBs are the major determinants of the kinetics of tension activation and of the early isometric phase of relaxation whereas the regulatory Ca^2+^ switch is a much faster process (Poggesi et al. 2005; Stehle et al. 2009). Some of the evidence collected during the last years in favour of the dominant role of CBs in myofibril tension kinetics are listed below.

**Fig. 2:**
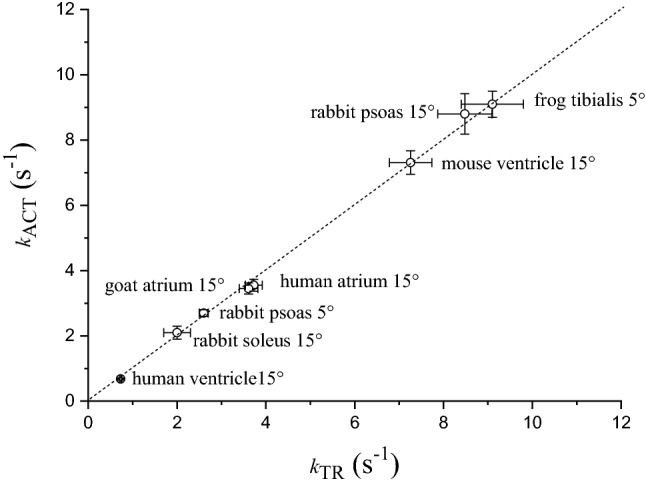
Rates of tension activation and tension redevelopment in different types of maximally Ca^2+^-activated skeletal and cardiac myofibril preparations at 5 or 15 °C. Mean ± SEM (n ≥ 11). Dotted line, identity line. Unpublished data
(ii) The lack of a significant influence of thin filament activation/inactivation kinetics on myofibril tension activation and relaxation kinetics was also clear from the comparison between fluorescence stopped flow studies on cardiac myofibril suspensions and mechanical studies on single cardiac myofibrils. The results (Solzin et al. 2007 reviewed by Stehle et al. 2009) showed that the structural changes in cardiac Troponin (Tn) following calcium binding to or dissociation from cardiac TnC in response to sudden increase or decrease in calcium (Tn switch) are much faster than myofibril force activation and relaxation kinetics.
(iii) Tn replacement studies in myofibrils further support the idea that contraction-relaxation myofibril kinetics, following abrupt increase/decrease in [Ca^2+^], are mainly set by the intrinsic CB cycling rate and are not directly altered by the kinetics of the Tn switch (Piroddi et al. 2003; de Tombe and Stienen 2007). Notably, as shown in Fig. 1 and Table 1, the kinetics of force activation and relaxation in mouse ventricular myofibrils are almost tenfold faster than in human ventricular myofibrils. However, exchange of human cardiac Tn for the endogenous mouse cardiac Tn in mouse ventricular myofibrils does not slow down force kinetics (see Table 1) indicating that it is the myosin motor form that sets the force kinetics of cardiac sarcomeres. Studies of replacement of all thin filament regulatory proteins (Tn and Tropomyosin) in myofibrils confirm the idea of a dominant role of CB cycling rate in determining the kinetics of sarcomere tension activation and relaxation (Siththanandan et al. 2009; Scellini et al. 2010).

(i) Under the conditions of our myofibril experiments (maximal Ca^2+^-activation, sarcomere length 2.20 µm, low [P_i_], relatively low temperature 5–15 °C) *k*_ACT_ and *k*_TR_ are essentially identical in a variety of preparations that largely differ in tension kinetics (Fig. 2). As already mentioned above, *k*_TR_ is measured under quasi-steady state conditions of thin filament activation and is thought to reflect CB turnover; *k*_ACT_ could in principle be a slower process as it may also be limited by thin filament activation kinetics. As shown in Fig. 2, *k*_ACT_ never falls below the identity line with *k*_TR_ strongly suggesting that both parameters primarily reflect CB turnover rate whereas Ca^2+^-activation is a much faster process.

**Table 1:** Kinetic parameters of mouse and human ventricular myofibrils following replacement by exchange of the endogenous cTn complex with human recombinant cTn

Myofibril type	*k*_ACT_ (s^−1^)	*k*_TR_ (s^−1^)	Slow *k*_REL_ (s^−1^)	Fast *k*_REL_ (s^−1^)
Mouse Ventr (control)	7.35 ± 0.54	7.12 ± 0.61	1.45 ± 0.21	28.3 ± 2.9
Mouse Ventr (human cTn exchanged)	7.05 ± 0.44	6.92 ± 0.75	1.55 ± 0.28	25.3 ± 3.1
Human Ventr (human cTn exchanged)	0.84 ± 0.03	0.78 ± 0.04	0.30 ± 0.02	4.65 ± 0.19

Data are mean ± SEM; n ≥ 10. Data from human cTn exchanged mouse myofibrils and control (sham-treated) mouse myofibrils are unpublished data; data from human cTn exchanged human ventricular myofibrils are from Piroddi et al. (2019). The exchange protocol allows replacement of 70–90% of the endogenous cTn complex
(iv) Changes in the concentrations of substrate and products of the acto-myosin ATPase markedly affect myofibril force activation and relaxation kinetics (Tesi et al. 2002). For instance, inorganic phosphate accelerates force kinetics in cardiac myofibrils; in particular it increases the rate of the slow force decay, consistent with the idea that slow *k*_REL_ reflects the dissociation of force generating CBs to non-force-generating states via both forward and backward transitions (Stehle and Tesi 2017). At variance with the large impact of interventions that interfere with key CB transitions, interventions that increase myofilament Ca^2+^-sensitivity by altering the Ca^2+^ Tn switch usually do not significantly affect *k*_ACT_, *k*_TR_, and slow *k*_REL_ measured in cardiac myofibrils at maximal activation (Piroddi et al. 2007; Kreutziger et al. 2011).

To better interpret myofibril tension kinetic parameters in terms of apparent CB kinetics we need a description of the reaction pathway for acto-myosin ATPase and energy-transduction cycle. The reaction pathway is usually described as a series of coupled biochemical and mechanical events with a relatively large number of transitions (Gordon et al. 2000; see also Fig. 5 in Ferrantini et al. 2009). Generally accepted schemes can be reduced for our purposes to a simpler two-state CB scheme (Brenner 1988). On the basis of myosin’s binding affinity for actin, two general types of CBs can be defined in the two-state model of acto-myosin interactions: AM_no force_ that represents all detached and “weak binding states” (detached states M.ATP, M.ATP.Pi in rapid equilibrium with AM.ATP, AM.ADP.Pi) and AM_force_ that represents all “strong binding states” (AM, AM*.ADP). The exoergonic release of inorganic phosphate (P_i_) is thought to power the working stroke. The transition from the non-force-generating states to the force generating states has an overall apparent rate constant *f*_*app*_. It is well known that at physiological [P_i_] the power stroke reverse transition may occur so that the return to the non force generating states may occur both through the backward transition (P_i_ rebinding and reversal of the power stroke) and the forward transition (ADP release and ATP binding). The apparent rate constant for the forward transition is *g*_app_ while the apparent rate constant for the reverse transition of the force generating step is *f′*_*app*_ that depends on [Pi].

$${\text{AM}}_{{{\text{No}}\,{\text{Force}}}} \mathop \rightleftarrows \limits_{{f^{\prime}_{app} }}^{{f_{app} }} {\text{AM}}_{\text{Force}} \mathop \to \limits^{{g_{app} }} {\text{AM}}_{{{\text{No}}\,{\text{Force}}}}$$

In the virtual absence of P_i_, that is the experimental condition of most myofibril studies, the apparent rate constant for the reverse transition *f′*_app_ can be neglected. Thus, during myofibril relaxation, following Ca^2+^ removal, strongly bound CBs can leave the force-generating states only via forward transitions whereas weakly-bound CBs are unable to go into the force-generating states (Poggesi et al. 2005; Ferrantini et al. 2009). In terms of CB turnover kinetics, myofibril slow *k*_REL_ measured in the absence of P_i_ consists solely of forward CB detachment, that leads to ATP hydrolysis steps, continuing at the same rate as during the isometric contraction; in short slow *k*_REL ~ _*g*_app_.

According to this model, the overall CB turnover rate, given by *f*_app_ + *g*_app_, is reflected by *k*_ACT_ and *k*_TR_, maximal tension is proportional to *f*_app_/(*f*_app_ + *g*_app_), ATPase activity is proportional to *f*_app_ · *g*_app_/(*f*_app_ + *g*_app_), and the energy cost of tension generation (ATPase/tension ratio) is proportional to *g*_app_ (Brenner, 1988; de Tombe and Stienen 2007). Given slow *k*_REL_ ≈ *g*_app_, an increase/decrease in slow *k*_REL_ should be able to predict an increase/decrease in the amount of ATP spent to generate a given amount of isometric force (i.e. the energy cost of tension generation).

Direct and coupled measurements of isometric tension and ATPase activity under isometric conditions can unequivocally confirm this conclusion.

### Cardiac sarcomere energetics and the correlation between energy cost of tension generation and the isometric relaxation rate of cardiac myofibrils from different preparations

Chemically permeabilized cardiac muscle strips, isolated from human and animal hearts, are the conventional preparations used to measure the isometric rate of ATP consumption of the sarcomeric proteins and to directly correlate the amount of ATP hydrolyzed to the amount of developed force (i.e. the tension cost of cardiac contraction) (de Tombe and Stienen 1995, 2007; Narolska et al. 2005; Witjas-Paalberends et al. 2014a, b). The removal of virtually all membrane structures leaves the sarcomeric proteins (i.e. myosin motors) as the only source of energy consumption in these preparations and this allows us to circumvent many of the problems that hamper myothermal measurements on intact myocardium (Holubarsch et al. 1991; Alpert et al. 1991; de Tombe and Stienen 2007). Indeed, interpretation of myothermal experiments in intact preparations can be mainly complicated by uncertainties in the relative amount of total energy that is used by the excitation contraction coupling system or by the sarcomeric proteins (Gibbs et al. 1988). It is to be noted, however, that in recent myothermal studies the use of blebbistatin, that selectively inhibits myosin activity, has allowed more accurate estimate of the energy expenditure associated with Ca^2+^ handling in intact cardiac muscle (Pham et al. 2017).

One of the main advantages of skinned preparations is that they allow standardization of the experimental conditions (e.g. composition of the intracellular medium, level of Ca^2+^-activation), thus minimizing disturbing factors present during the normal twitch of intact cardiac muscle (i.e. variable calcium concentration, hormonal factors, concentrations of metabolic products) (van der Velden et al. 1998; Narolska et al. 2005). The simultaneous measurement of isometric force and ATPase activity in skinned cardiac strips, by means of an enzymatic coupled assay, allows a direct correlation between mechanic and energetic properties of cardiac sarcomeres.

**Fig. 3:**
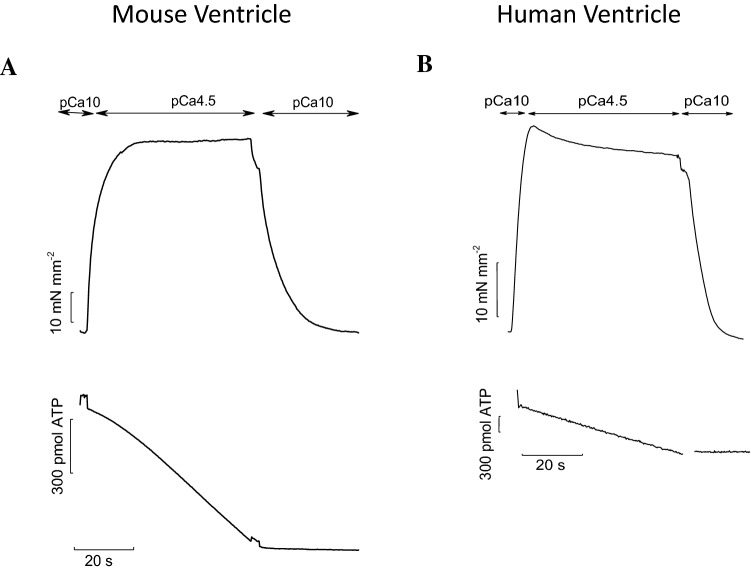
Simoultaneous measurements of isometric tension and ATPase activity in permeabilized mouse and human ventricular strips. Representative traces of force (top) and ATPase activity (bottom) from permeabilized mouse (**A**) and human (**B**) ventricular strips at maximal Ca^2+^-activation. The muscle strips were activated in saturating [Ca^2+^] solution (pCa 4.5). Isometric force started to develop and NADH absorbance signal started to decline. After force and the slope of the absorbance signal had reached a steady level the muscle strips were relaxed in a low [Ca^2+^] solution (pCa10). The rate of ATP consumption was determined from the slope of the NADH absorbance signal during the last 20 s of activation. Notably, the slope is much steeper in mouse ventricle strip (**A**) than in human preparation (**B**), indicative of a greater ATP consumption to develop force. Sarcomere length 2.2 µm. Temperature: 20 °C

In this method, the re-synthesis of ATP, catalyzed by pyruvate kinase, is enzymatically coupled to the oxidation of nicotinamide dinucleotide (NADH), catalyzed by lactate dehydrogenase. The breakdown of NADH can be determined photometrically by measuring the absorbance of 340-nm near-UV light (Glyn and Sleep 1985; Stienen et al. 1993; Kentish and Stienen 1994; Potma et al. 1994; de Tombe and Stienen 1995, 2007). As shown in Fig. 3, simultaneous measurements of maximal isometric tension and ATPase activity in mouse and human ventricular strips at optimum overlap demonstrate that the slope of the NADH absorbance signal during steady activation is markedly higher in the mouse versus the human preparation. The difference in the amount of maximal tension developed by the two preparations is not enough to account for the higher ATPase found in the mouse myocardium. This indicates that tension cost is much greater in the mouse sarcomeres.

**Fig. 4:**
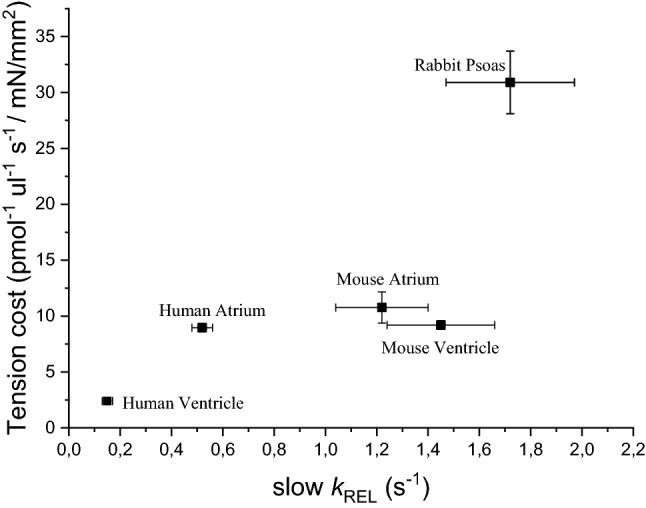
Relation between slow *k*_REL_ and tension cost of different striated muscle preparations. There is a significant correlation (r = 0.90) between slow relaxation kinetics of myofibrils (15 °C) and the tension cost of larger permeabilized preparations (20 °C) from a variety of striated muscle tissues in spite of potential differences in the experimental conditions used in the different laboratories were the measurements were taken. Tension cost data of human atrial and ventricular preparations are from Narolska et al. (2005); slow *k*_REL_ data for the myofibrils from human atrial and ventricular samples are from Piroddi et al. (2007). Tension cost data of rabbit psoas (consistent with earlier measurements), are from Joumaa et al. (2017); slow *k*_REL_ data for the myofibrils from the same muscle are from Kreutziger et al. (2008). Tension cost and slow *k*_REL_ data for skinned mouse ventricular trabeculae and myofibrils are from Ferrantini et al. (2017). Tension cost and slow *k*_REL_ data for skinned mouse atrial trabeculae and myofibrils are unpublished data from the Authors’ laboratory. All data are shown as mean ± SEM

By combining mechanical experiment on single myofibrils (Fig. 1) and simultaneous mechanic and energetic experiments on larger skinned muscle preparations (Fig. 3) one can directly investigate the relation between CB turnover rate and ATP utilization during isometric muscle contraction. We found that, in disparate types of striated muscles from different species, the slow *k*_REL_ measured in myofibrils in our laboratory significantly correlated with the respective energy cost of isometric tension generation measured by different laboratories in skinned preparations from the same muscle type (Fig. 4). In spite of the difference in temperature between the experiments in myofibrils and multicellular skinned preparations and the potential differences in the experimental conditions used in the different laboratories where the energetic measurements were taken the correlation is rather good. Tension cost data in Fig. 4 are from Narolska et al. (2005) (human atrial and ventricular muscle), from Joumaa et al. (2017) (rabbit psoas), from Ferrantini et al. (2017) (mouse ventricle), and from unpublished data from our laboratory for mouse atrial muscle.

The correlation in Fig. 4 is strong evidence in support of the idea that slow *k*_REL_ ≈ *g*_app_ ≈ tension cost. Of note, the positive correlation between these two parameters holds true for muscle types that greatly differ in their actomyosin ATPase rate. In human myocardium, the slow β Myosin Heavy Chain (β-MHC) isoform is predominantly expressed in the ventricles whereas the fast α-MHC isoform is the main molecular motor of the atria. Previous studies on rat and rabbit samples had shown that the fast α-MHC isoform exhibits a two to three times higher actin-activated ATPase activity (Pope et al. 1980) and actin filament sliding velocity than the slow β-MHC isoform (Harris et al. 1994). We also had previously found that the much faster relaxation kinetics of atrial vs ventricular myofibrils are consistent with the large difference in CB kinetics between the two MHC isoforms (Piroddi et al. 2007). The proportionality between CB kinetics and tension cost is maintained, since direct measurements of ATPase activity confirm that a ~ fourfold higher slow *k*_REL_ (Piroddi et al. 2007) corresponds to a ~ fivefold higher tension cost in atrial vs ventricular human myocardium (Narolska et al. 2005). At variance with human myocardium, in rodents’ heart α-MHC is the main myosin isoform expressed both in the atria and in the ventricles. Of note, as shown in Fig. 4, the kinetics of the slow phase of relaxation and tension cost are rather similar between preparations from the two cardiac chambers of the mouse.

### Cardiomyopathy-related alterations of CB kinetics and energetics: increased slow relaxation kinetics underlie higher tension cost

Myofibril kinetics and energetics have been shown to be altered by several sarcomeric protein mutations associated to genetic cardiomyopathies. In particular, HCM is a genetic disease primarily caused by mutations in the genes encoding sarcomeric proteins (e.g. for reviews see Watkins et al. 2011; Poggesi and Ho 2014). To date, it is unclear how these mutations lead to the cardiac phenotype also because patients carrying the same causal mutation may exhibit different clinical phenotypes.

With the exception of truncation mutations in cardiac myosin binding protein C for which evidence of haploinsufficiency has been generated (Marston et al. 2009; van Dijk et al. 2009), most HCM mutations result in stable proteins that are incorporated into the sarcomere and may act as poison peptides on the contractile performance, (e.g. Cuda et al. 1993; Bottinelli et al. 1998). In vitro studies on human and animal HCM muscle preparations and isolated proteins suggested that the pathogenic impact of the mutations can be attributed to different mechanisms such as: aberrant CB dynamics leading to decreased/increased contractility, (e.g. Cuda et al. 1993; Palmiter et al. 2000; Belus et al. 2008; Palmer et al. 2008; Witjas-Paalberends et al. 2014a), increased intrinsic force of the myosin motor (Seebohm et al. 2009; Sommese et al. 2013), increased sarcomeric Ca^2+^ sensitivity, e.g. (Bottinelli et al. 1998, for a review see Marston 2011). To reconcile the lack of consistent contractility changes in HCM, it has been proposed that HCM sarcomere mutations may lead to increased energy cost of force generation through inefficient or excessive ATPase activity. This results in an energy deficiency of cardiomyocytes that ultimately contributes to the pathogenesis of the disease (Ashrafian et al. 2003). Several studies support this pathogenic hypothesis (Jung et al. 1998; Crilley et al. 2003; Javadpour et al. 2003; Chandra et al. 2005; He et al. 2007; Luedde et al. 2009; Witjas-Paalberends et al. 2014b).

Investigations of the impact of some HCM-related mutations on human cardiac myofibrils revealed a marked acceleration of the apparent rate with which force-generating CBs detach under isometric conditions (Belus et al. 2008; Ferrantini et al. 2009; Witjas-Paalberends et al. 2014a; Ferrantini et al. 2017; Piroddi et al. 2019; Vitale et al. 2019). These studies suggested that a primary mutation-driven effect on myofilaments can be an increase in tension cost. Direct measurement of ATPase activity in multicellular muscle strips from patients confirmed that the energy cost of isometric tension development was higher in HCM patients carrying specific sarcomeric protein mutations compared to controls and to HCM patients without an identified sarcomeric gene mutation (HCM_smn_, sarcomere mutation negative). This was true both for mutations of thick filament proteins, such as the R403Q myosin mutation, and for mutations of thin filament proteins, such as the K280N cardiac TnT mutation. While the marked impact of the R403Q mutation in myosin on CB kinetics and sarcomere energetics may be not surprising, the mechanisms of the effects of the K280N mutation in cTnT are much less clear. Pioneer studies, reviewed by Gordon et al. (2000), reported that HCM-associated cTnT mutations may cause changes in regulated acto-S1 ATPase and unloaded thin filament sliding speed. This implies that cTnT can modulate the CB kinetics of the strongly bound state in addition to its ability to control the attachment of CBs to the thin filament.

**Fig. 5:**
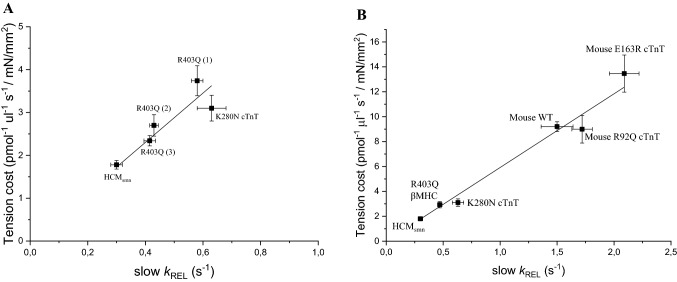
Relation between slow *k*_REL_ and tension cost in ventricular samples from human and mouse HCM models. All data are shown as mean ± SEM. **A** Data from human ventricular samples from different HCM patients. The correlation between slow relaxation kinetics of human ventricular myofibrils (15 °C) and tension cost of human myocardial strips (20 °C) can be described by a line with slope 5.75 (r = 0.99). Data from three patients carrying the R403Q mutation in β-myosin heavy chain [indicated as R403Q (1), (2), and (3)] and from HCM patients without an identified sarcomeric gene mutation (sarcomere mutation negative indicated as HCM_smn_) are from Witjas-Paalberends et al. 2014a. Data from the patient carrying a homozygous mutation in cTnT (K280N) are from Piroddi et al. 2019. **B** Data from human and mouse models. The correlation between slow relaxation kinetics of ventricular myofibrils (15 °C) and tension cost of myocardial strips (20 °C) can be described by a line with slope 5.92 (r = 0.99). Human data are the same as in panel A with the individual data of the 3 R403Q patients replaced by the average data from all 3 patients. Mouse data are taken from Ferrantini et al. (2017). Independently from species, mutations, and disesase state, slow *k*_REL_ measured in myofibrils correlates linearly with the tension cost measured in multicellular muscle strips from the same samples

As shown in Fig. 5A, in all human HCM patient samples analyzed so far (three R403Q beta-MHC, one K280N cTnT, a few sarcomere mutation negative patients) it can be observed that slow *k*_REL_ varies in proportion with the respective energy cost of tension development of each individual human cardiac sample independently from the protein carrying the mutation or even in the absence of any mutations (Fig. 5A). Data in Fig. 5A are replotted from published data (Witjas-Paalberends et al. 2014a; Piroddi et al. 2019).

An altered economy of muscle contraction associated to a faster rate of isometric relaxation in myofibrils was also found in an HCM mouse model carrying a mutation (E163R) of cTnT (Ferrantini et al. 2017). In the same study it was also shown that a second mouse model carrying a different mutation in the same protein (R92Q cTnT) did not exhibit the biophysical phenotype described above. As shown in Fig. 5B, a clear correlation between slow *k*_REL_ and tension cost is maintained for all human and mouse models investigated independently from species, mutations, and disease states. The slope of the linear relation is relatively high and essentially the same as found in Fig. 5A. The same linear relation is maintained for changes in tension cost and slow *k*_REL_ of one order of magnitude. Mouse data in Fig. 5B are from Ferrantini et al. (2017); human data are the same as in Fig. 5A with the individual data of the 3 R403Q patients replaced by the average data from all 3 patients.

## Conclusions and implications

Overall, the data reviewed here provide evidence that a clear linear proportionality exists between the isometric apparent detachment rate of CBs (slow *k*_REL_) and the energetic cost of tension generation of the sarcomere measured in preparations from the same muscle sample. The relationship holds true among a variety of striated muscles from different species and also when CB kinetics is altered by sarcomeric mutations, strengthening the expectation of the simple two-state CB model (Brenner 1988) that the kinetics of CB detachment under isometric conditions along forward transitions (g ~ slow *k*_REL_) correlates linearly with the amount of ATP spent per unit force.

The experimental observations that sarcomeric protein mutations associated to HCM are often associated to increased tension cost (Fig. 5) may, in principle, suffer from artifacts due to the structural remodeling often undergone by cardiac muscle in HCM. In fact, cardiomyocyte disarray may artificially decrease isometric tension and increase the “isometric” ATPase of multicellular preparations resulting in artificial increase in the tension cost. To address the problem we have developed techniques to reconstruct the 3D structure of the whole ventricular strips used for mechanical and energetic experiments (Vitale et al. 2019). Following 3D image reconstruction of the strips at sub-micrometer spatial resolution, cardiomyocyte orientation across and along the strips is determined. Statistics of spatial disarray are derived and correlated to mechanical and energetic data. Preliminary results (Vitale et al. 2019) do not highlight structural differences between donor and HCM strips strengthening the conclusion that HCM mutations primarily alter apparent CB kinetics and impair sarcomere energetics.

Is the increase in the energy cost of isometric tension generation, observed with a number of HCM-associated mutations, expected to alter the overall cost of the actual cardiac cycle? Though our data do not say much about the energetic cost of contraction under mechanical conditions that are not isometric, the specific impact of a number of mutations associated to HCM on isometric CB kinetics and isometric tension cost may help explaining some yet unresolved clinical features of HCM. Generation of large amounts of isometric tension in the heart only occurs in the walls of the left ventricle (LV), especially during isovolumic contraction, though high tension is also maintained during LV ejection. Though the mutant proteins may be expressed in all cardiac chambers, the disease specifically affects the LV causing an asymmetric hypertrophy most often affecting the interventricular septum. Theoretical models challenge the assumption of uniform myocardial wall tension in the LV contraction (DeAnda et al. 1998) and indicate the septum as the region where the highest stress is generated. This may be also supported by studies that show greater energy demands in the septum (Dunn 1984). Superimposing regional mechanical and metabolic differences on HCM myocytes might lead to localized uncompensated energetic load and hence asymmetric hypertrophy.

Does the increased energy cost of tension generation and the consequent energy impairment of HCM cardiomyocytes contribute to the asymmetric LV hypertrophy observed in HCM? Using magnetic resonance spectroscopy, a reduction in the cardiac PCr to ATP ratio, a measure of energetic status, has been reported in HCM patients with LV hypertrophy (Crilley et al. 2003). In the same study, abnormal mean PCr/ATP ratios have also been found in pre-hypertrophic carriers of HCM mutations (genotype positive/clinical phenotype negative subjects) in which the energetic alterations cannot be attributed to the confounding hypertrophy. Therefore, it can be proposed that a primary stimulus for hypertrophy may be the energetic defect itself. If energy generation cannot match demand, cytosolic Ca^2+^ re-uptake/removal at the end of each contraction is compromised, particularly due to the extreme energy requirements of SERCA2, the sarcoplasmic reticulum Ca^2+^ re-uptake pump. The resultant prolonged cytosolic Ca^2+^ transient may be able to trigger downstream hypertrophy pathways.
